# Knowledge, Attitude, and Performance of ICU, CCU, and Emergency Wards Nurses in Kermanshah, Iran, regarding Organ Donation

**DOI:** 10.1155/2020/5167623

**Published:** 2020-09-27

**Authors:** Maryam Janatolmakan, Ali Soroush, Roghayeh Nouri, Bahare Andayeshgar, Alireza Khatony

**Affiliations:** Clinical Research Development Center, Imam Reza Hospital, Kermanshah University of Medical Sciences, Kermanshah, Iran

## Abstract

**Background:**

Providing an organ for donation is a major problem worldwide and nurses play an important role in facilitating the process of organ donation. This study is aimed at investigating the knowledge, attitude, and performance of nurses working in the ICU, CCU, and emergency wards regarding organ donation.

**Methods:**

In this descriptive-analytical study, 185 nurses working in ICU, CCU, and emergency wards were studied through systematic random sampling. The data collection was done by a self-administered questionnaire.

**Results:**

The mean knowledge of nurses was 8.9 ± 1.4 out of 10. There was a significant relationship between knowledge of nurses regarding donation and religion and having organ donation card (*P* < 0.001). The mean attitude of nurses was 7.8 ± 2.2 out of 8. The variables, including “having a donation card and marriage,” were associated with attitude of nurses toward organ donation. The mean performance of nurses was 0.4 ± 0.7 out of 3. There was a significant relationship between performance of nurses and having a donation card (*P* < 0.001). Knowledge was the strongest predictor of nurses' performance (*P* < 0.01).

**Conclusion:**

The studied nurses showed sufficient knowledge and favorable attitude toward organ donation; however, they had poor performance. It is suggested to hold training courses to improve performance of nurses. The revision of the nursing students' curriculum as future nurses should also be considered.

## 1. Introduction

Organ donation and organ transplants are social activities used to survive and improve the lives of millions of people who have the chance to live [1, 2]. Organ failure has adverse effects on the quality of life of people, which results in mortality. Despite several advancements in medicine, organ transplant is the only treatment option for those with End-stage organ failure [3, 4]. However, many patients with organ failure die due to organ shortage for transplantation [5]. Today, the demand for organ transplants is increasing worldwide with an increasing number of those in the waiting list for organ transplantation [3, 6, 7]. Iran is considered as one of 30 countries with high demand for organ donation [8]. Accordingly, every 10 minutes, one person is added to the waiting list for organ transplant, and, every two hours, one patient dies while waiting for organ transplantation. In Iran, however, one person is dead every 70 minutes due to brain death [9]. Organ failure is a major concern worldwide, so promoting organ donation in all countries is essential [10]. Although organ donation is a personal issue, its process is complex and multidimensional, including medical, legal, ethical, organizational, and social aspects [10, 11]. Nurses are regarded as the key factors in facilitating the organ donation process, which can help prevent the loss of transplantable organs by obtaining family consent for organ donation. Their knowledge and attitude regarding organ donation can influence public opinion as well as the relatives' decision about deceased organ donation [1, 12]. In the United Kingdom, the organ donation process is primarily supervised by nurses, and identifying potential organ donors is one of their duties [5]. In this regard, the results of a study in Korea showed that 69% of the donors with transplantable organs were introduced by nurses [1]. Several factors, including religion, education, work experience, age, the relationship with the deceased's family, a history of caring for brain-dead patients, and personal experiences have positive or negative impacts on the attitude of nurses toward organ donation [7, 9, 10, 13]. Facilitating the organ donation process should be considered as one of the nurses' professional responsibilities, and as they provide nursing management for patients with traumatic injuries, the criteria for brain death should be assessed by nurses and they also should identify organ donors [4]. Inadequate knowledge of nurses regarding the legal details of organ donation as well as organ donation procedures can be one of the important reasons for the low number of donors. Therefore, increasing the knowledge of nurses regarding the rules of organ donation is crucial [10–12]. Several studies have examined the knowledge and attitude of nurses regarding organ transplantation [5, 11, 14]. According to a study in Sweden, the performance of nurses working in ICU was significantly associated with the increase or decrease of the number of organ transplants [13]. The results of a study in Ireland indicated that some nurses were not aware of the process of organ donation as well as the diagnosis of brain death [15]. In a study in India, about two-thirds of nursing students had sufficient knowledge and a positive attitude toward organ donation; however, 94% of the subjects were not aware of the rules regarding organ donation [11]. The results of a study in China showed that 33.4% of nurses were willing to donate their organs after their death [12]. In a study in Pakistan, only 35% of healthcare workers showed willingness to donate their organs [16].

Therefore, to facilitate organ donation, it is necessary to evaluate the knowledge and attitude of nurses regarding organ donation, since they are directly connected with organ donors and they may affect patients' families in the decision-making process [5]. Due to the important role of nurses in facilitating the organ donation process and lack of knowledge, attitude, and performance of nurses working in Kermanshah University of Medical Sciences, this study aimed to investigate the factors associated with knowledge, attitude, and performance of nurses working in ICU, CCU, and emergency wards regarding organ donation.

## 2. Materials and Methods

### 2.1. Study Design

This cross-sectional study was conducted from May to July 2019.

### 2.2. Study Questions

In this study we sought to answer the following questions. (1) How much do nurses know regarding organ donation? (2) How is the attitude of nurses regarding organ donation? (3) How is the performance of nurses regarding organ donation? (4) What is the relationship between knowledge of nurses regarding organ donation and age, gender, work experience, a history of caring for brain-dead patients, nurses with a donation card, having donation card in nurses' families, education, and religion? (5) What is the relationship between the attitude of nurses regarding organ donation and age, gender, work experience, a history of caring for brain-dead patients, nurses with a donation card, having donation card in nurses' families, education, and religion? (6) What is the relationship between performance of nurses regarding organ donation and age, gender, work experience, a history of caring for brain-dead patients, nurses with a donation card, having donation card in nurses' families, education, and religion? (7) What is the relationship between knowledge, attitude, and performance of nurses?

### 2.3. Sample and Sampling Method

The study population consisted of 450 nurses working in ICU, CCU, and emergency wards of the hospitals affiliated to Kermanshah University of Medical Sciences (eight hospitals). The sample size was estimated to be 185 subjects using Cochran formula with 95% confidence and 0.05 error and *P*=0.55. Inclusion criteria included bachelor's degree or above in nursing, working in one of the ICU, CCU, and emergency wards, and at least one year of clinical experience. Samples were selected by systematic random sampling. Accordingly, the names of nurses were obtained from the nursing offices of the hospitals and coded. Then, using a randomized table, 185 nurses were selected through systematic random sampling.

### 2.4. Measurement Instrument

A two-part questionnaire was used for data collection. The first part included eight questions assessing personal information, such as age, sex, work experience, a history of caring for brain-dead patients, having a donation card, having donation card in nurses' families, education, and religion. The second part was the organ donation questionnaire developed by Chakradhar et al., in which the knowledge, attitude, and performance of nurses regarding organ donation is assessed [17]. It has been psychometrically evaluated in Iran by Purbahram et al. in 2017 [18]. In the present study, the validity of the questionnaire was determined by content validity. For this purpose, 12 faculty members were provided with the questionnaire and their comments were included in the questionnaire. The internal consistency of the questionnaire was assessed by Cronbach's alpha (*α* = 0.76).

The Chakradhar questionnaire is a 27-item scale, including 13 questions on knowledge (questions 1 to 13), 11 questions on attitude (questions 14 to 24), and 3 questions on performance (questions 25 to 27). It is completed by yes/no questions, which are scored with one and zero, respectively. The answer to all questions is yes, except for questions 6, 9, 10, 21, and 23. The obtained scores from the knowledge, attitude, and performance domains are ranged between 0–10, 0–8, and 0–3, respectively. The higher scores represent higher levels of knowledge, attitude, and performance of the nurses. In the present study, the performance of nurses means having a history of receiving or donating an organ or having an organ donation card, which was determined by answering questions 25 to 27.

### 2.5. Data Collection Method

After obtaining approval from the University Ethics Committee, the researcher referred to the selected nurses according to their work schedule. The participants were informed about the research objectives. Written informed consent was obtained from all of them. Then, the questionnaires were provided to the participants, and after completion, it was collected.

### 2.6. Data Analysis

The data were analyzed by the Statistical Package for Social Sciences (SPSS v.18.0; SPSS Inc., Chicago, IL, USA) using descriptive and analytical statistics. Shapiro–Wilk test was used to assess the normality of quantitative variables, including knowledge, attitude, and performance of nurses, in which results indicated the normal distribution of these variables. Stepwise linear regression was used to identify the predictors of nurses' knowledge, attitude, and performance regarding organ donation. Stepwise Multiple Linear Regression was used to predict the most powerful variables in order to predict the knowledge, attitude, and performance of nurses regarding organ donation. The significance level was *P* < 5% for all tests.

### 2.7. Ethical Considerations

The research was approved by the University Ethics Committee (ethics code: IR.KUMS.REC.1398.101). All participants were informed of the research objectives and their questions were answered. All participants were assured of the confidentiality of their information. The written informed consent was obtained from all participants.

## 3. Results

About half of the samples (49.7%, *n* = 92) were in the age range of 23–33 years, with a mean age of 33.9 ± 7.2 years. Most of subjects were female (72.4%, *n* = 134), were married (58.4%, *n* = 108), and had a bachelor's degree (82.7%, *n* = 153). Of the evaluated samples, 104 nurses (56.2%) reported formal employment in nursing (87%, *n* = 161). All samples (*n* = 185, 100%) were Muslim and majority of cases were Shia Muslims (*n* = 154, 83.2%). Regarding work experience, 79 cases (42.7%) had 1–8 years of work experience with the average work experience of 10.2 ± 6.8 years. Of the evaluated cases, 27.6% (*n* = 51) of the nurses and 21.6% (*n* = 40) of their families had organ donation cards. A history of caring for brain-dead patients was reported by 143 subjects (77.3%) (Table 1).

The mean knowledge of nurses was 8.9 ± 1.4 out of 10 (Table 2). Stepwise Multiple Linear Regression was used to predict the strongest variables to predict nurses' knowledge regarding organ donation. Nurses who had organ donation cards were significantly more aware of organ donation. Besides, Sunni nurses were also more aware than Shia nurses. The results showed that, in the first step, “having a donation card” as the strongest predictor alone predicted 5% of the variance in knowledge of nurses (*F* = 10.67, *P* < 0.001). In the second step, the religion as the second variable was included in the prediction of knowledge of nurses regarding organ donation. Including religion increased the prediction through organ donation card and religion to 8.9% (*F* = 9.93, *P* < 0.001). The results showed that no other predictor variable is included in the next step of prediction (Table 3).

The mean attitude of nurses was 7.8 ± 2.2 out of 8 (Table 2). The results of Stepwise Multiple Linear Regression showed that variables, including “having organ donation card and marital status,” were associated with nurses' attitude toward organ donation, as those with organ donation cards had a better attitude toward organ donation compared to the nurses without a card. Married nurses also had a better attitude toward organ donation than single nurses. In the Stepwise method, organ donation card as the strongest predictor alone predicts 3.6% of the variance of nurses' attitude toward organ donation (*F* = 7.80, *P*=0.006). In the second step, the marital status as another strong predictor was included in the prediction of nurses' attitude following having a donor card variable. Including marital status increased predictive value to 5.8% (*F* = 6.67, *P*=0.002). Multiple regression results showed that no other predictor could enter the model in the next step (Table 4).

The mean performance of nurses was 0.4 ± 0.7 out of 3 (Table 2). The results of Stepwise Multiple Linear Regression showed that only “having a donation card” had a significant effect on the performance of nurses. In the first step, “having a donation card” as the strongest predictor alone predicted 2.98% of the variance of nurses' performance (*F* = 79.26, *P* < 0.001). The results showed that no other predictor could enter the model (Table 5).

**Figure 1:**
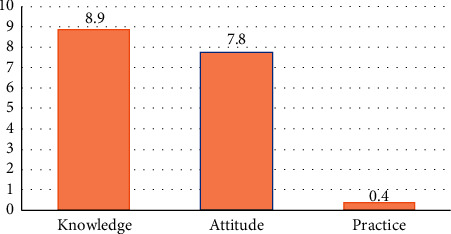
The mean of nurses' knowledge, attitude, and practice regarding organ donation.

According to the results of Stepwise Multiple Linear Regression, nurses' knowledge had a significant effect on their performance. In the first step, knowledge as the strongest predictor alone predicted 2.58% of the variance of nurses' performance (*F* = 6.56, *P* < 0.01). The results showed that no other predictor could enter the model (Table 6) (Figure 1).

## 4. Discussion

Nurses play an important role in organ donation and organ transplantation. Their knowledge and attitude can improve people's reactions to organ donation and prevent their resistance to organ donation [12]. In this regard, the present study aimed to investigate the knowledge, attitude, and performance of nurses working in ICU, CCU, and emergency wards regarding organ donation.

In our study, nurses showed a desirable level of knowledge regarding organ donation. According to a study in India, 66.7% of nurses had sufficient knowledge about organ donation [11]. The results of a study on Korean nurses indicated that they had desirable level of knowledge regarding organ donation [5]. In another study in Turkey, healthcare workers had enough knowledge about organ donation [19]. Given the academic nature of the nursing profession and the training provided to nurses during their education, they are expected to have desirable knowledge regarding organ donation.

In the present study, there was a significant relationship between knowledge of nurses regarding organ donation, religion, and having a donation card, since the Sunni nurses had more knowledge about organ donation. These results are in line with the results of other relevant studies [17, 20].

The results of a study on knowledge, attitude, and performance of dental students regarding organ donation in India showed a significant relationship between religion and knowledge of students regarding organ donation, as those Christians, Jains Atheists' nurses were shown a higher level of knowledge about organ donation than other religions [17]. In another study entitled “Knowledge, Attitude, and Performance of Adults regarding Organ Donation in South India,” a significant relationship was found between knowledge about organ donation and religion, as the Hindu population was more aware of organ donation [20]. Uncertainty about the religious aspects of organ donation can be one of the obstacles in the organ donation process. In Islam, organ transplant is altruistic, and it has been confirmed by many great religious scholars in Islamic countries. In this regard, explaining religious instruction regarding organ transplant and organ donation through CME courses can increase nurses' awareness.

In the current study, there was a significant relationship between nurses' knowledge about organ donation and having an organ donation card and nurses who were more aware of organ donation had an organ donation card, which is consistent with the results of other studies [21, 22]. In this regard, the results of a study in Mashhad, Iran, showed that nurses who had organ donation cards were more aware of organ donation [21]. In another study in Tehran, Iran, there was a direct correlation between nurses' knowledge and organ donation card [22]. Therefore, having sufficient knowledge about the organ donation process is an important factor in persuading nurses to have an organ donation card. In this respect, nurses as those who are interested in the community can be effective in culture-making of having an organ donation card and also play an important role in increasing organ donation in the community [23].

The results showed that nurses' attitude toward organ donation was relatively favorable. In the results of a study on the attitudes of Chinese nurses toward organ donation, only 33.4% were willing to be organ donors [12]. The result of another study in India indicated a positive attitude of nursing students toward organ donation [11]. The findings of Araujo and Siqueira study in Brazil indicated that about 90% of healthcare workers were willing to donate [1].

According to our results, there was a significant relationship between nurses' positive attitude toward organ donation, marital status, and having a donation card, so that married subjects had a favorable attitude toward organ donation. In a study aimed at assessing the knowledge and attitude of Korean nurses toward organ donation of brain-dead patients, married nurses had a better attitude toward organ donation [5]. However, in a study conducted in Brazil aimed at examining attitudes of health workers toward organ donation, no significant relationship was found between attitudes toward organ donation and marital status [1]. The family is regarded as one of the bases for changes in values in society. Marriage and family formation can also be associated with new values and play a decisive role in creating people's attitudes.

According to the results of the present study, there was a significant relationship between positive attitude toward organ donation and having an organ donation card, which is consistent with other relevant studies. In this regard, the results of a study in Iran aimed at examining the knowledge and attitude of nurses toward organ donation showed that there is a significant relationship between positive attitude toward organ donation and having an organ donation card [22]. In a systematic review study, aimed at evaluating the awareness and role of healthcare workers regarding organ donation and transplants, staff who had organ donation card had a favorable attitude toward organ donation [24]. In another study conducted in Tehran, Iran, among 78% of physicians who had a positive attitude about organ donation, 68% had organ donation cards [25]. In contrast, a study in India showed that although 81% of nurses were willing to donate organs after their death, only 3.8% had organ donation cards [26]. In a study in Turkey aimed at examining medical students' knowledge, awareness, and attitudes toward organ donation, there was no significant relationship between attitude and having a donation card and despite the positive attitude of half of the samples, only 2% had a donation card [27]. Attitude can make people ready for doing a particular behavior and the more positive attitude results in the increased likelihood of doing the behavior.

In the present study, performance of nurses regarding organ donation was undesirable. The result of a study done in India showed poor performance of dental students toward organ donation [17] The results of a study on Indian nursing students' knowledge, attitude, and readiness regarding organ donation showed that most of the subjects had poor performance and only a limited number of them had organ donation card [11], which is consistent with our results. Proper measures should be taken to improve performance of nurses regarding organ donation and transplants and regular training courses can also be effective. In addition, a revision of the nursing education curriculum is essential to prepare students, as future nurses for organ donation and transplants. In this regard, the result of a study in Mashhad, Iran, showed that nurses with organ donation cards showed favorable performance regarding organ donation [21], which is not consistent with our results. The results of a study in India indicated that 42.3% of the subjects had sufficient knowledge about organ donation cards and only 3.7% of them had the card [28]. Adequate knowledge and a proper attitude are the requirements to create a behavior. Although, in our study, nurses showed favorable knowledge and attitude toward organ donation, they were found with poor performance in organ donation. Poor institutionalization of organ donation can be considered as one of the reasons for the poor performance of nurses.

According to the results of our study, nurses' knowledge had a significant effect on their performance. Accordingly, those with sufficient knowledge in organ donation demonstrated appropriate performance. The attitude and performance of nurses are expected to be improved by increasing their knowledge.

The current research was a cross-sectional study, in which it is not possible to derive causal relationships between the study variables. Data collection was done by a self-administered questionnaire, which can affect the accuracy of the results.

## 5. Conclusion

Nurses working in ICU, CCU, and emergency wards had sufficient knowledge and attitudes toward organ donation, but they were found with poor performance. Moreover, nurses' knowledge was also a strong predictor for their performance regarding organ donation. Healthcare providers are recommended to take measures to improve nurses' performance in organ donation by providing appropriate training programs. In this regard, holding continuous education courses for nurses on the organ donation process is of particular importance. Besides, assigning a full-time nurse to follow up on organ donation-related issues can be helpful. Further studies on nurses working in other wards and also investigating the impact of interventional measures on the level of knowledge, attitude, and performance of nurses regarding organ donation process and organ transplants are suggested.

## Figures and Tables

**Table 1:** Demographic characteristics of nurses (*n* = 185).

Variables		No (%)
Age (year)	23–33	92 (49.7)
	34–43	76 (41.1)
	44–56	17 (9.2)

Sex	Male	51 (27.6)
	Female	134 (72.4)

Marital status	Single	77 (41.6)
	Married	108 (58.4)

Education	BSc.	153 (82.7)
	MSc.	32 (17.3)

*y*.card^*a*^	Yes	51 (27.6)
	No	134 (72.4)

*F*.card^*b*^	Yes	40 (21.6)
	No	134 (72.4)
	I do not know	11 (5.9)

Religion	Shia	154 (83.2)
	Sony	31 (16.8)

Work experience (year)	1–8	79 (42.7)
	9–17	76 (41.1)
	18–25	30 (16.2)

Caring experience of brain-death patient	Yes	143 (77.3)
	No	42 (22.7)

^*a*^Nurses' donation card. ^*b*^Family's donation card.

**Table 2:** Nurses' mean knowledge, attitude, and practice in terms of demographic variables.

Variables		Knowledge mean ± SD^*a*^	Attitude mean ± SD	Practice mean ± SD
Age (year)	23–33	9.01 ± 1.37	7.56 ± 2.21	0.51 ± 0.77
	34–43	8.80 ± 1.36	8.05 ± 2.14	0.34 ± 0.57
	44–56	8.88 ± 1.31	8.29 ± 2.25	0.41 ± 0.79

Sex	Male	8.90 ± 1.43	7.94 ± 2.23	0.49 ± 0.78
	Female	8.91 ± 1.34	7.79 ± 2.18	0.41 ± 0.67

Marital status	Single	8.93 ± 1.31	7.36 ± 2.38	0.45 ± 0.77
	Married	8.89 ± 1.39	8.16 ± 1.98	0.41 ± 0.65

Education	BSc.	8.87 ± 1.38	7.73 ± 2.28	0.39 ± 0.70
	MSc.	9.09 ± 1.27	8.28 ± 1.65	0.59 ± 0.71

Self.card^*b*^	Yes	9.43 ± 1.26	8.54 ± 1.60	1.05 ± 0.42
	No	8.71 ± 1.35	7.55 ± 2.32	0.19 ± 0.64

*F*.card^*c*^	Yes	9.07 ± 1.30	8.17 ± 2.44	0.65 ± 0.69
	No	8.79 ± 1.38	7.67 ± 2.14	0.33 ± 0.68
	I do not know	9.72 ± 1.10	8.45 ± 1.57	0.818 ± 0.75

Religion	Shia	8.79 ± 1.36	7.92 ± 2.11	0.44 ± 0.68
	Sony	9.48 ± 1.23	7.38 ± 2.52	0.35 ± 0.79

Work experience (year)	1–8	8.93 ± 1.35	7.65 ± 2.31	0.51 ± 0.76
	9–17	8.90 ± 1.45	7.92 ± 2.08	0.30 ± 0.61
	18–25	8.86 ± 1.19	8.06 ± 2.16	0.53 ± 0.73

Caring experience of brain-death patient	Yes	9.02 ± 1.35	7.97 ± 2.11	0.44 ± 0.69
	No	8.54 ± 1.34	7.35 ± 2.39	0.40 ± 0.73

Total		8.91 ± 1.36	7.83 ± 2.19	0.43 ± 0.70

^*a*^Standard deviation, ^*b*^nurses' donation card, and ^*c*^family's donation card.

**Table 3:** Factors affecting nurses' knowledge of organ donation.

Step	Predictive variable	Beta	*t*	*P*-value	*R* ^2^	Adjusted *R*^2^	*F*	*df*	*P*-value
1	Self.card^∗^	0.71	3.26	0.001	0.055	0.050	10.67	1,183	0.001

2	Self.card	0.76	3.56	<0.001	0.098	0.089	9.93	2,182	<0.001
	Religion (sony)	−0.71	−2.95						

^∗^Nurses' donation card.

**Table 4:** Factors affecting nurses' attitude regarding organ donation.

Step	Predictive variable	Beta	*t*	*P* value	*R* ^2^	Adjusted *R*^2^	*F*	*df*	*P* value
1	Self.card^∗^	0.98	2.79	0.006	0.041	0.036	7.80	1,183	0.006

2	Self.card	0.92	2.63	0.009	0.068	0.058	6.67	2,182	0.002
	Marital status	0.73	2.31	0.022					

^∗^Nurses' donation card.

**Table 5:** Factor affecting nurses' performance regarding organ donation.

Step	Predictive variable	Beta	*t*	*P* value	*R* ^2^	Adjusted *R*^2^	*F*	*df*	*P* value
1	Self.card*∗*	0.55	8.90	<0.001	0.302	0.298	79.26	1,183	<0.001

^∗^Nurses' donation card.

**Table 6:** The impact of nurses' knowledge on their organ donation performance.

Step	Predictive variable	Beta	*t*	*P* value	*R* ^2^	Adjusted *R*^2^	*F*	*df*	*P* value
1	Acknowledge	0.186	2.58	0.011	0.35	0.029	6.56	1,183	0.011

## Data Availability

The data used in the study are available upon reasonable request.

## References

[1] Araujo C., Siqueira M. (2016). Brazilian healthcare professionals: a study of attitudes toward organ donation. *Transplantation Proceedings*.

[2] Babaie M., Hosseini M., Hamissi J., Hamissi Z. (2015). Knowledge, attitude and practice of nurses regarding organ donation. *Global Journal of Health Science*.

[3] Soylar P., Kadioğlu B. U. (2018). Theology and nursing students’ knowledge of organ donation and transplantation. *Transplantation Proceedings*.

[4] Majeed F. (2016). Saudi nursing and medical student’s knowledge and attitude toward organ donation—a comparative cross-sectional study. *International Journal of Health Sciences*.

[5] Choi J. Y., Ko J. W., Park M. R. (2016). Hospital nurses’ knowledge and attitude toward brain-dead organ donation. *International Journal of Bio-Science and Bio-Technology*.

[6] Kinge A., Bhate K., Yadav A., Vyas P. (2017). Knowledge and concerns for organ donation amongst nursing students and nursing staff in an apex medical institute in a Metropolitan City. *Journal of Evolution of Medical and Dental Sciences*.

[7] Vlaisavljević Z., Janković S., Soldatović I. (2017). Survey of knowledge and attitudes of head nurses regarding organ transplantation. *Acta Medica Medianae*.

[8] Bahrami A., Khaleghi E., Khorsand-Vakilzadeh A., Afzalaghaee M. (2017). Process and barriers to organ donation and causes of brain death in northeast of Iran. *Electronic Physician*.

[9] Safari-Moradabadi A., Madani A., Zare F., Amani F., Dadipoor S. (2014). Awareness and attitude of Bandar Abbas residents towards organ donation. *Iranian Journal of Health Education and Health Promotion*.

[10] Abbasi M., Kiani M., Ahmadi M., Salehi B. (2018). Knowledge and ethical issues in organ transplantation and organ donation: perspectives from Iranian health personnel. *Annals of Transplantation*.

[11] Poreddi V., Katyayani B., Gandhi S., Thimmaiah R., Badamath S. (2016). Attitudes, knowledge, and willingness to donate organs among Indian nursing students. *Saudi Journal of Kidney Diseases and Transplantation*.

[12] Xie J.-F., Wang C.-Y., He G.-P. (2017). Attitude and impact factors toward organ transplantation and donation among transplantation nurses in China. *Transplantation Proceedings*.

[13] Forsberg A., Lennerling A., Fridh I., Rizell M., Lovén C., Flodén A. (2015). Attitudes towards organ donor advocacy among Swedish intensive care nurses. *Nursing in Critical Care*.

[14] Pelicic D., Vukcevic B., Bokan D., Stojanovic V., Radojevic N. (2019). Attitudes toward organ donation and transplantation among transplant-related health care workers and the local population of Montenegro. *Experimental and Clinical Transplantation*.

[15] Lomero M. d. M., Jiménez-Herrera M. F., Rasero M. J., Sandiumenge A. (2017). Nurses’ attitudes and knowledge regarding organ and tissue donation and transplantation in a provincial hospital: a descriptive and multivariate analysis. *Nursing & Health Sciences*.

[16] Siddiqui O. T., Nizami S., Nizami S. (2012). Deceased-donor organ transplantation: knowledge and attitudes among health care professionals managing critically ill patients in Karachi. *Experimental and Clinical Transplantation*.

[17] Chakradhar K., Doshi D., Srikanth Reddy B., Kulkarni S., Padma Reddy M., Sruthi Reddy S. (2016). Knowledge, attitude and practice regarding organ donation among Indian dental students. *International Journal of Organ Transplantation Medicine*.

[18] Purbahram R., Ashktorab T., Barazabadi Farahani Z., Nasiri M. (2017). Knowledge and attitude of the intensive care unit nurses in mazandaran province towards organ donation. *Iran Journal of Nursing*.

[19] Demir T., Selimen D., Yildirim M., Kucuk H. (2011). Knowledge and attitudes toward organ/tissue donation and transplantation among health care professionals working in organ transplantation or dialysis units. *Transplantation Proceedings*.

[20] Sarveswaran G., Sakthivel M. N., Krishnamoorthy Y., Arivarasan Y., Ramakrishnan J. (2018). Knowledge, attitude, and practice regarding organ donation among adult population of urban Puducherry, South India. *Journal of Education and Health Promotion*.

[21] Hoseini S. M., Manzari Z., Khaleghi I. (2015). ICU nurses’ knowledge, attitude, and practice towards their role in the organ donation process from brain-dead patients and factors influencing it in Iran. *International Journal of Organ Transplantation Medicine*.

[22] Aghayaw H. R., Arjmand B., Emami-Razavi S. H. (2009). Organ donation workshop—a survey on nurses’ knowledge and attitudes toward organ and tissue donation in Iran. *The International Journal of Artificial Organs*.

[23] Salmela S., Eriksson K., Fagerström L. (2012). Leading change: a three‐dimensional model of nurse leaders’ main tasks and roles during a change process. *Journal of Advanced Nursing*.

[24] Jawoniyi O., Gormley K., McGleenan E., Noble H. R. (2018). Organ donation and transplantation: awareness and roles of healthcare professionals—a systematic literature review. *Journal of Clinical Nursing*.

[25] Nadoushan M. S., Heshmati B. N., Pirsaraee A. S., Nodoushan I. S., Nadoushan R. J., Yazdi F. (2014). Knowledge and attitude of Iranian physicians towards organ and tissue donation. *International Journal of Organ Transplantation Medicine*.

[26] Vijayalakshmi P., Nagarajaiah R., Ramachandra S. B., Math S. B. (2015). Indian ICU nurses’ perceptions of and attitudes towards organ donation. *British Journal of Nursing*.

[27] Kocaay A., Celik S., Eker T., Oksuz N., Akyol C., Tuzuner A. (2015). Brain death and organ donation: knowledge, awareness, and attitudes of medical, law, divinity, nursing, and communication students. *Transplantation Proceedings*.

[28] Mithra P., Ravindra P., Unnikrishnan B. (2013). Perceptions and attitudes towards organ donation among people seeking healthcare in tertiary care centers of coastal South India. *Indian Journal of Palliative Care*.

